# Interactive exploration of integrated biological datasets using context-sensitive workflows

**DOI:** 10.3389/fgene.2014.00021

**Published:** 2014-02-20

**Authors:** Fabian Horn, Martin Rittweger, Jan Taubert, Artem Lysenko, Christopher Rawlings, Reinhard Guthke

**Affiliations:** ^1^Systems Biology/Bioinformatics, Leibniz Institute for Natural Product Research and Infection Biology - Hans Knöll InstituteJena, Germany; ^2^Department of Computational and Systems Biology, Rothamsted ResearchHarpenden, UK

**Keywords:** exploratory analysis, Ondex, data integration, data visualization, information network, *Aspergillus nidulans*, customizable workflow, gold-standard

## Abstract

Network inference utilizes experimental high-throughput data for the reconstruction of molecular interaction networks where new relationships between the network entities can be predicted. Despite the increasing amount of experimental data, the parameters of each modeling technique cannot be optimized based on the experimental data alone, but needs to be qualitatively assessed if the components of the resulting network describe the experimental setting. Candidate list prioritization and validation builds upon data integration and data visualization. The application of tools supporting this procedure is limited to the exploration of smaller information networks because the display and interpretation of large amounts of information is challenging regarding the computational effort and the users' experience. The Ondex software framework was extended with customizable context-sensitive menus which allow additional integration and data analysis options for a selected set of candidates during interactive data exploration. We provide new functionalities for on-the-fly data integration using InterProScan, PubMed Central literature search, and sequence-based homology search. We applied the Ondex system to the integration of publicly available data for *Aspergillus nidulans* and analyzed transcriptome data. We demonstrate the advantages of our approach by proposing new hypotheses for the functional annotation of specific genes of differentially expressed fungal gene clusters. Our extension of the Ondex framework makes it possible to overcome the separation between data integration and interactive analysis. More specifically, computationally demanding calculations can be performed on selected sub-networks without losing any information from the whole network. Furthermore, our extensions allow for direct access to online biological databases which helps to keep the integrated information up-to-date.

## 1. Introduction

In our study, we developed and applied customizable context-sensitive menus to the data integration and visualization tool Ondex. This allows for the interactive exploration of experimental data that is integrated into an information network. The introduction starts with a survey of network inference methods and qualitative assessment of inferred networks. Exploratory data analysis looks for new patterns and hypothesis in a dataset and it is thus well suited to qualitatively assess network modeling and experimental results within the context of an information network.

### 1.1. Qualitative assessment of network inference

Network inference reconstructs molecular interaction networks on the basis of experimental high-throughput data. Nodes in the resulting network usually represent molecular entities (e.g., genes or proteins), for which concentration or activity has been measured using omics-technology. The edges in the network stand for direct and indirect relationships between the molecular entities, i.e., they symbolize diverse modes of regulation or direct molecular interaction. New molecular relationships may be predicted with the help of network inference modeling techniques. The predictions are new biological hypotheses which result from the given experimental data. A highly diverse variety of network inference modeling techniques have been developed based on differential equation systems or Bayesian networks (as reviewed in Hecker et al., 2009). Each modeling technique utilizes a wide range of modeling parameters which are optimized mainly on the given experimental data. For example, the gene regulatory network inference method NetGenerator (Guthke et al., 2005; Töpfer et al., 2006; Weber et al., 2013) is based on differential equation systems which minimizes both the model fit error, i.e., the difference between the measured and the simulated data of time-series experiments, and the number of model parameters. Additionally, prior knowledge is used to guide the inference process (Linde et al., 2010, 2012).

In order to validate the chosen modeling technique and its parameter optimization, it is necessary to assess the validity of the resulting biological networks. Quantitative measures utilize an error model and model selection criteria, e.g., least square error model and Akaike's Information Criterion, which makes use of the experimental data and the inferred model (Rao et al., 2008). The internal validation evaluates whether the model is robust and can be generalized. For these purposes, subsampling (cross-validation), bootstrapping and network perturbations are applied (reviewed by Hecker et al., 2009). Another aspect is the utilization of benchmark data which can be either generated from an artificially constructed gene regulatory network or the experimental data is gathered from a well-researched biological system. As an example, the DREAM challenge provided gene expression data from a synthetically generated network which consisted of five genes (Cantone et al., 2009).

**Network Inference**

- Q: How can exploratory analysis be used for the validation of inferred networks?
- A: It needs to be qualitatively assessed if inferred network components describe the experimental setting. Tools like Ondex provide automatic data integration and visualization which facilitate exploratory data analysis as well as the quality control.
- Q: What are the challenges for this kind of qualitative network validation?
- A: The large amount of available information leads to a high computational effort during the data integration and the automatic data visualization. This may result in a non-satisfying users' experience.
- Q: What method is introduced to overcome these limitations?
- A: We introduce the concept of context-sensitive workflows for Ondex. During the data exploration, it allows for the integration of additional information for a set of interesting features. Thus, computational-demanding calculations are only performed for a subnetwork, which greatly improves the usability of the tool for network validation.

Nevertheless, the parameters of each network inference technique cannot be optimized based on experimental or simulated data alone. The particular outcome of a network reconstruction needs to be qualitatively assessed by verifying that its components describe the experimental setting and that they are in accordance to prior knowledge. For this step, test data (“gold-standard”) is required, which was not included in the training dataset for the network inference. It consists of expert knowledge and data which was predicted with the help of bioinformatic tools, e.g., the software tool SiTAR for transcription factor binding sites predictions (Fazius et al., 2011). As a second aspect, network inference methods can infer genome-wide networks which may contain thousands of nodes and relations (Altwasser et al., 2012). Validation of these genome-wide networks is hard because the number of model parameters is very high and the gold-standard used is usually too small to make generalizations about the quality of the whole network. Due to the large size of the resulting network, the experimental validation with a high quality standard is not suitable. Thus, all components and proposed interactions need to be interpreted and prioritized before further experimental analysis.

### 1.2. Exploratory data analysis

From a methodological point of view, feature selection or candidate prioritization can be performed by two complementary approaches: exploratory and confirmatory data analysis (Tukey, 1977, 1980). Confirmatory data analysis starts with an open, precise question. Usually, it is a fixed procedure and it is, hence, especially suited to be performed by a computer, e.g., using statistics or guided pathway exploration. In contrast, exploratory data analysis (as introduced by Tukey, 1980) does not follow a direct route between question and answer, but allows for iterative cycles between research question, experimental design and gathered data. Computer analysis is needed to search for the right questions and hidden relationships buried within the massive amounts of data coming from high-throughput methods. Another aspect is that data stored within public data repositories usually has been analyzed with respect to a limited number of research questions. Therefore, there is still a considerable potential to gain new insights from this data, but the challenge is to find the right questions in order to perform a successful meta-analysis or re-analysis of the data. Despite the importance of exploratory analysis for research, very few software systems are available that support the requirements to integrate multiple sources of biological data and provide the rich set of analysis methods needed for exploratory data analysis (discussed in Kelder et al., 2010). To help researchers recognize patterns within the data more readily and to enable them to concentrate on the interpretation of the data, software tools should perform all automatable tasks of handling large data amounts, i.e., the data integration and automatic data visualizations.

### 1.3. Ondex—a software solution for exploratory analysis

Many software tools have been developed for data integration and visualization using network structures. [A good review of data integration methodologies and tools is given by Huttenhower and Hofmann (2010) and Bebek et al. (2012) and tools for visualization of biological network data are described by Pavlopoulos et al. (2008).] In this study, we use the Ondex data integration framework, which combines data integration, analysis, and visualization (Köhler et al., 2006). While Ondex shares many of its features with other tools, its main advantage lies in its flexible data representation and available visualization methods (Taubert et al., 2007). It is very suitable for exploratory data analysis—meaning the exploration of experimental data without prior hypotheses and a pre-defined data analysis workflow. Ondex uses a graph-based core data structure where nodes represent biological entities (e.g., genes or proteins) and edges represent the relationships between them (e.g., “a gene *encodes* a protein”). Using ontologies, Ondex automates the integration of heterogeneous data from diverse sources (e.g., structured data repositories, flat files, or free-text) into a semantically consistent graph representation. The provenance of the data is retained during the integration process. The modular plug-in architecture of Ondex enables the addition of extra functionality, such as parsers for new data sources or complex filtering methods. The Ondex front-end facilitates interactive visualization, searching, and filtering of the datasets. Certain attributes contained in the graph can be associated with the color, glyphs, and visibility of nodes and edges. Ondex is open source, written in platform-independent Java and supports open standards and interfaces. The Ondex data integration framework has already successfully been used for the study of microarray expression data (Köhler et al., 2006), data integration for plant genomics (Lysenko et al., 2009), supporting *in silico* drug discovery (Cockell et al., 2010), and finding genes implicated in plant stress response (Hassani-Pak et al., 2010).

Despite the advantages and the successful application of Ondex, the handling of large amounts of data is still challenging. The system can be used to produce integrated datasets with several millions of entries, which makes efficient querying and visualization difficult. Additionally, the data warehousing approach of Ondex means that some of the data can become out-of-date. This contrasts with the approach used by federated data integration systems, which always query live data resources [e.g., Taverna (Hull et al., 2006)]. To overcome these limitations to some extent, the Ondex front-end already offers the possibility to iteratively explore parts of the graph and link-out to more recent data available in online resources.

In this paper, we present the implementation of interactive context-sensitive workflows in Ondex to improve the analysis of large integrated datasets.

### 1.4. Our work

We applied Ondex to construct a gold-standard information network for *Aspergillus nidulans* as a basis for the qualitative assessment of reconstructed networks. The network inference for filamentous fungi is challenged by the circumstance that prior knowledge is limited and widely scattered. It needs to be collected from literature and diverse databases, or predictions need to be made with the help of additional bioinformatic tools (Horn et al., 2012). As a consequence, no extensive knowledge network exists which can function as gold-standard. *A. nidulans* is the main model organisms for filamentous fungi and substantial knowledge about the regulation of secondary metabolites exists (Brakhage, 2013). Secondary metabolites may directly contribute to the pathogenicity of fungi, e.g., gliotoxin was found to modulate the immune response and induce apoptosis in cells of *A. fumigatus* (Scharf et al., 2012). The knowledge about the mechanisms of regulation of secondary metabolites of *A. nidulans* can be transferred to other filamentous fungi, especially if filamentous fungi share the same secondary metabolite gene clusters. As an example, the penicillin gene cluster is present in *A. nidulans* and *Penicillium chrysogeneum*. Generally, most fungal gene clusters are silent under standard laboratory conditions, and it is promising for drug target research to systematically determine conditions under which these gene clusters are expressed and secondary metabolites are produced (Walton, 2000; Brakhage and Schroeckh, 2011). Prominent examples are non-ribosomal peptide synthetases (NRPS) and polyketide synthases (PKS), which are two main classes of secondary metabolites that often serve as drug lead structures (Newman and Cragg, 2012). Most gene clusters are currently not functionally annotated (Sanchez et al., 2012) making the investigation of gene clusters challenging.

One of our objectives was to facilitate simultaneous interactive data integration during visual data exploration. This procedure has been implemented and is available for the community (see section 2). In order to demonstrate the practical relevance of our approach, the context-sensitive menus and the invocation of external web services were applied to the integrated network for *A. nidulans*. This also included data from an expression profiling experiment comparing the wild-type and a ΔcnsE-mutant at different developmental stages (Nahlik et al., 2010).

## 2. Materials and methods

In this section, we present (1) an information network for *A. nidulans* and (2) an extension of the Ondex framework in the form of context-sensitive menus, which are subsequently (3) used to analyze a transcriptome experiment. In this section, the functionality of the menus and generalizable workflows and approaches are presented. Exploration strategies which are based on the specific data, intermediate results, and the research question of the experiment are presented in the results section. The workflow of data integration, the resulting network, and the context-specific menu items are available from http://ondex.rothamsted.ac.uk/anidulans.

### 2.1. Data integration: an application case for *Aspergillus nidulans*

**Figure 1:**
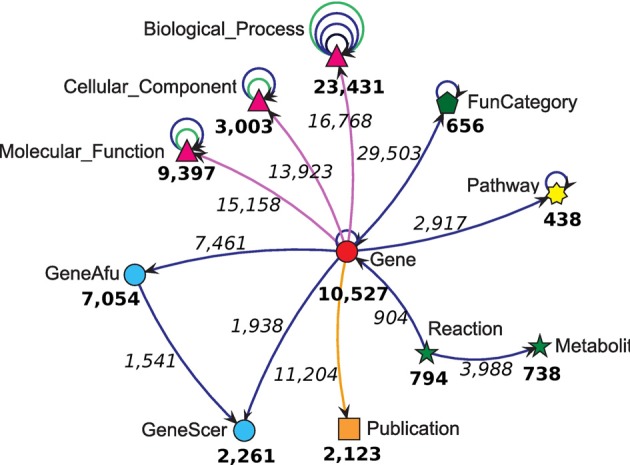
**Meta-legend of the integrated dataset for *A. nidulans* depicting all available concept classes**. The number of concepts within a concept class is given in bold numbers, whereas the number of mappings or relations between respective concept classes are given in italic numbers.

Experimentally-derived data for most fungi is scarce, incomplete, and scattered over several resources. Additionally, this data undergoes rapid changes due to newly assigned annotations and new genome assemblies. We integrated several publicly available datasets for *A. nidulans* using the pre-existing plug-ins from the Ondex integrator. (An overview of the data sources used and the extracted data is given in Table 1 and Figure 1). *Gene* concepts are mapped to the Gene Ontology hierarchies (Ashburner et al., 2000) (*Biological_Process, Cellular_Component, Molecular_Function*). Additional functional annotation data from the FunCat (*Functional Categories*) (Ruepp et al., 2004) and KEGG Pathways (*Pathway*) (Kanehisa et al., 2012) was integrated. In order to allow comparative analysis of *A. nidulans*, orthologous gene mappings to *Aspergillus fumigatus* and *Saccharomyces cerevisiae* were included. A mapping between publications and genes was performed if these genes were in the focus of the publication. A list of manually-curated publications was downloaded from the Aspergillus Genome Database (AspGD) (Arnaud et al., 2010). Additionally, a metabolic network (David et al., 2008), reflecting the regulatory relationships of enzymatic reactions by regulatory genes was integrated.

**Table 1:** **Data sources for *A. nidulans* information network**.

**Data source**	**Web address**	**Description**	**Concept class**
Aspergillus genome database	www.aspgd.org	Gene ontologies	GO
		Homologues	GeneAfu, GeneScer
		Literature	Publication
		Synonyms	Gene
Gene ontology	www.geneontology.org	Gene ontologies	GO
Ensembl fungi (CADRE)	http://fungi.ensembl.org	Annotation	Gene
		Chromosomal position	Gene
		Identifier mapping	Gene
KEGG (version: 2011)	www.genome.jp/kegg/	Pathways	Pathway
MIPS functional catalog	http://pedant.gsf.de	FunCat ontologies	FunCat
David et al. (2008)	www.biomedcentral.com	Metabolic network	Metabolite, reaction
GEO (GSE22442) (Nahlik et al., 2010)	www.ncbi.nlm.nih.gov/geo/	Expression values	Gene

Several data sources for *A. nidulans* were parsed and mapped into one Ondex information network. Data was downloaded from several sources. Instead of using publicly available transcriptome data from GEO, in-house experimental data may be mapped.

Experimental data from a study from Nahlik et al. (2010) was integrated. This data is available from the Gene Expression Omnibus (GEO identifier: GSE22442). The focus of this experiment was to investigate the impact of the COP9 signalosome complex on the transcriptome. Two genotypes (wild-type and Δ*csnE*-mutant) were compared under four different induced growth stages—vegetative 14 h, vegetative 20 h, sexual 48 h, asexual 48 h. For each growth stage, two biological replicates each with four technical replicates were measured, summing up to a total of 32 samples. The raw data was downloaded from GEO and the biological replicates were normalized individually with the help of loess and quantile normalization provided by the limma package (Smyth and Speed, 2003). A preceding analysis of variance (ANOVA) showed that the highest systematic variation arises from the biological replicates rather than any other experimental source of variation. Thus, the signals were modeled independently with the linear model provided by the limma-package. Calculated *p*-values were corrected for multiple testing using the method by Benjamini and Hochberg (Benjamini and Yekutieli, 2001). Results from both biological replicates were combined using the z-transformation of the *p*-values suggested by Stouffer (Whitlock, 2005). Probe sequences were mapped to gene definitions of the *A. nidulans* structural genome annotation (Horn et al., 2011). According to this mapping, the experimental data was integrated by Ondex into the *A. nidulans* information network. In order to emphasize the level of regulation, the glyphs of the gene concepts were scaled and colored by Ondex according to the expression values of the corresponding transcripts. For each growth-stage, the resulting information network was further explored separately in order to adequately understand the underlying interactions and correctly interpret the experimental data with respect to the experimental setting.

### 2.2. Ondex extension: context-sensitive menus

**Figure 2:**
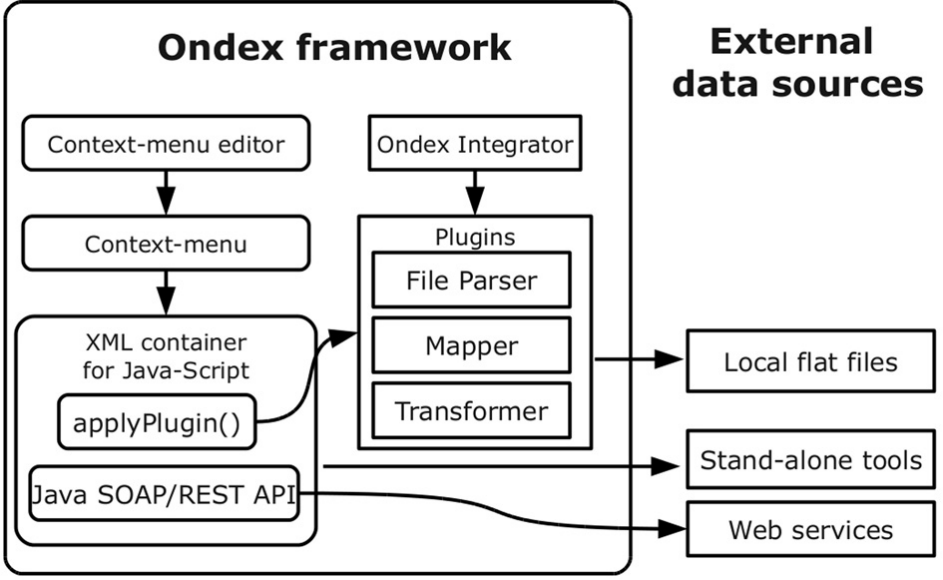
**Simplified modular view of context menu extension**. The new Ondex framework extension realizes context-sensitive menus which can be edited with the help of a GUI based editor. Concept specific entries are implemented in XML-files that contain Java Script code. Existing plug-ins for data integration from the Ondex library can be directly invoked. Web services and local installations of external programs can be called from the context menu.

The flexibility and power of the analysis offered by the Ondex system is realized primarily through the notion of customisability, i.e., users are free to build their own application cases from a set of generic re-usable components. Larger integration and analysis tasks are realized as workflow components, whereas the less substantial ones can be completed by calling a set of in-built functions. To that end, the Ondex system incorporates a JavaScript API (based on Mozilla Rhino v1.7) and a rich selection of binding and analysis functions that can be used both to manipulate the graph and to alter its appearance in the Ondex front-end. The binding and functions available via the scripting environment abstract some of the complexity of the Java-based Ondex API and allow for more concise syntax and greater convenience. This additional simplification is made possible by the use of run-time bytecode code generation (powered by the JavaAssist v3.12.0 library) that creates a set of wrappers. This setup allows both easy incorporation of additional external libraries and their seamless integration into the Mozilla Rhino scripting environment by automating the process of creating wrappers that implement additional interface(s) or delegate calls to multiple classes. The Ondex scripting environment can be accessed interactively using a console environment. In this work, we have extended this functionality further by developing a system of context-specific menus that can dispatch calls to the Ondex scripting APIs. The use of temporary sub-graphs also allows users to define their own JavaScript functions to be added as entries on these menus. For this study, we provide new functions for on-the-fly data integration using InterProScan, Blast, and PubMedCentral full-text search (see next section for more details). Using the modular architecture of Ondex, we extended the framework with context-sensitive pop-up menus which allow integration to be performed on the fly, while the network is being explored visually (see Figure 2). Throughout the paper, we keep the official nomenclature that nodes in an Ondex network are referred to as *concepts* (Taubert et al., 2007). The term *gene concept* hence describes one particular node which represents a gene entity. While examining the graph, users can integrate additional data or perform computationally demanding calculations for selected concepts. The menus are sensitive in regard to the concept class. This means that certain operations are only available for certain concept classes, e.g., BLAST operations are only available if the concept is a gene and contains sequence information. Internally, computational operations are either performed directly on the main graph or a temporary sub-graph, which initially consist only of the user-selected concepts. This mechanism also facilitates the re-use of the wide variety of Ondex workflow plug-ins, as individual workflow modules can also be called via particular items in the menu. Context menu functionalities are implemented using JavaScript code and they are stored as Extensible Markup Language (XML) files on the local file system using JavaBeans. Each XML-file represents one context menu item which can be restricted to be applicable only to the nodes of particular Ondex concept classes. The context-specific menus can also be organised hierarchically, where the nesting of the sub-menus is represented by the structure of the directories containing the XML-files in the file system.

The XML-files can either be edited with the help of external tools or with an embedded JavaScript editor. The graphical user interface (GUI) of the editor provides an easy way to specify concept class restrictions, integration of additional Java libraries and syntax highlighting with help of jEdit. The Ondex framework extension has been integrated into the main Ondex project and is freely available at http://www.ondex.org.

### 2.3. Specific workflow and customized context-sensitive menus for *Aspergillus nidulans* dataset

In Ondex, the precise workflow of exploration depends specifically on the integrated data, the research question, and the preferences of the user. Nevertheless, we performed analyses with a generally applicable workflow that gave us a first overview of the information contained in the data, i.e., filtering down to specific genes, biological processes, and underlying interactions. For our study, we provide new functionalities to Ondex through context-sensitive menus, namely the InterProScan, a sequence-based homology search, and a full-text literature search at PubMed Central. During the procedure described above, the log-fold changes of gene expression data from 32 samples were integrated as attributes to the gene concepts in the information network. This allowed us to significantly reduce the number of concepts (i.e., genes and proteins) by applying a filter based on the log-fold change and *p*-value. Thus, the number of interesting concepts which need to be manually checked were reduced. At the same time, all available information can be reconsidered during the analysis by redisplaying previously filtered data. We independently analyzed each contrast, i.e., the differences in transcript abundance between the Δ*csnE*-mutant and the wild-type at different time points (i.e., growth stages), and filtered for either differentially expressed transcripts (DEGs) (|fold-change| ≥ 4 and adjusted *P*-value ≤ 0.05) or for *strongly* differentially expressed transcripts (|fold-change| ≥ 8 and adjusted *P*-value ≤ 0.05). Previously integrated information, i.e., concepts such as gene ontologies and publications, was included in the visualization if it is associated with the resulting gene sets.

**Figure 3:**
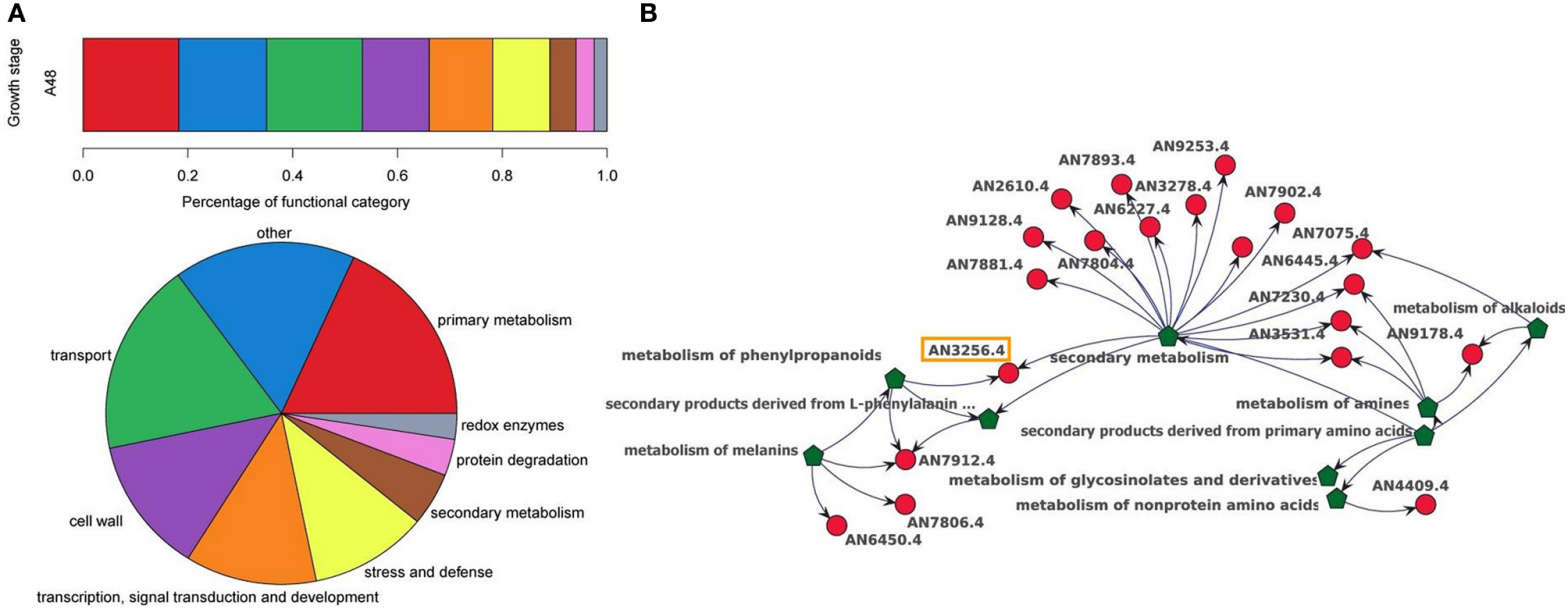
**Different visualization types of the distribution of functional annotations**. **(A)** Bar and pie chart diagram representing the percentage of prominent biological processes within the strongly differentially expressed genes (|fold-change| ≥8, adjusted P-value ≤0.05). Example charts are shown for the asexual developmental growth stage after 48 h (A48). **(B)** A detail of the sub-network representing the mapping between functional categories and strongly differentially expressed genes during the sexual development after 48 h. Green polygonic concepts represent gene ontology concepts, whereas red circular concepts represent genes. An arrow between two gene ontologies reflects the hierarchical structure of the directed acyclic graph of the gene ontologies. An arrow pointing from an ontology concept to a gene concept shows which genes have been assigned to the respective gene ontology concept. In contrast to commonly used pie and bar chart diagrams, the visualization within Ondex allows to explore possible multiple mappings between genes and ontologies without losing the information about the level of detail at which the mapping occurs.

The DEGs were subject to further exploratory analysis and the integrated dataset was used to identify which of the functional categories were predominantly up or down-regulated. For this purpose, only gene concepts which are differentially regulated were made visible and all connected gene ontology concepts have been visualized while retaining their hierarchical network structure (see Figure 3). The functional categories and their associated differentially expressed genes were arranged using a hierarchical layout (see Figure 3B). In order to make our results comparable to the publication from Nahlik et al. (2010), we additionally grouped and named our functional categories according to the terminology adopted by that paper (For details see Supplementary File S1). Ondex automatically sorts the networks by its size, i.e., the number of connected concepts. Thus, it is immediately possible to identify and further explore the annotation-orientated sub-networks where many DEGs have been mapped.

**Figure 4:**
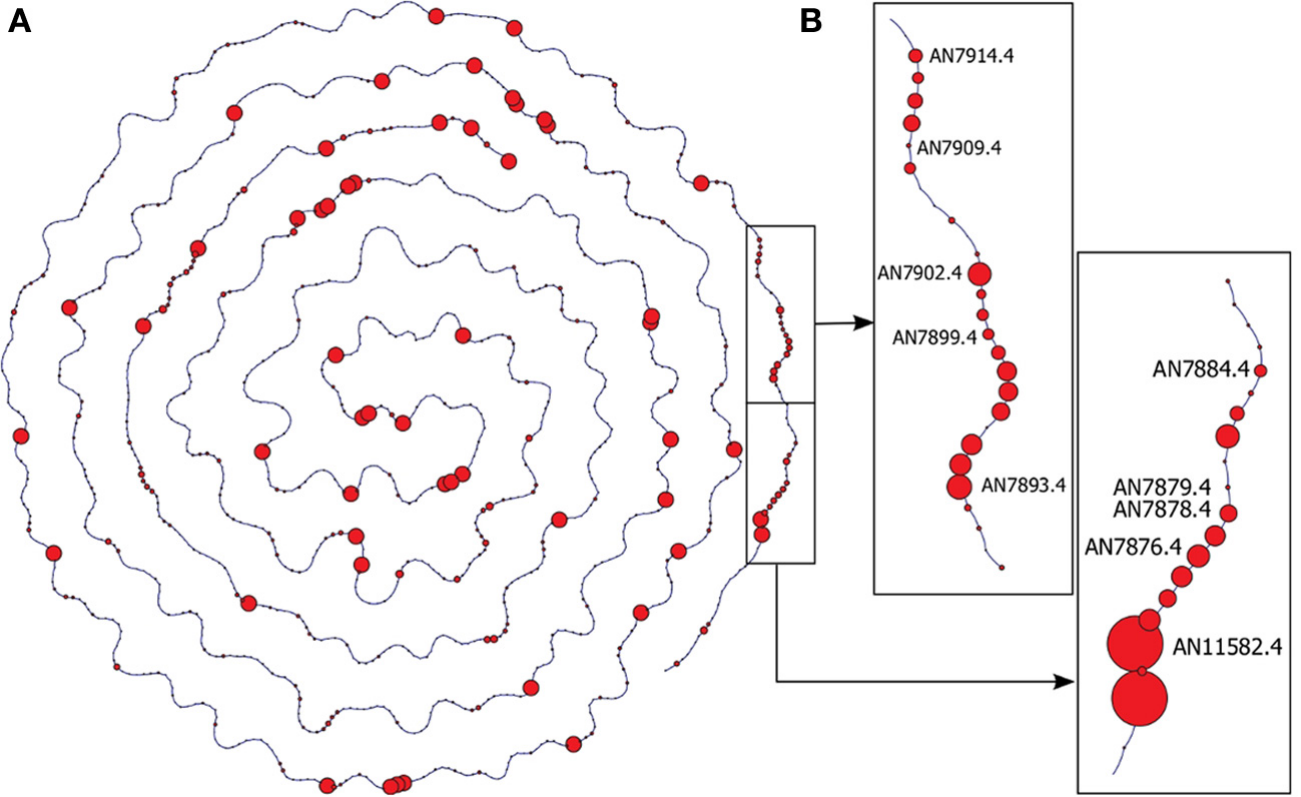
**Chromosomal View/Gene Cluster View**. **(A)** Gene concepts located near to each other on the chromosome are connected. Transcriptome data has been integrated and glyphs are scaled accordingly, i.e., the circle size represents the expression values of the corresponding transcripts. Regions with several strongly differentially expressed genes are possible indications for secondary metabolite gene clusters. The figure depicts chromosome II of *A. nidulans* with the transcriptomic data of the asexual developmental growth stage after 48 h (A48). **(B)** Detailed view of the neighborhood of two differentially expressed gene clusters: the orsellinic gene cluster (AN7909–AN7914) and a putative, uncharacterized gene cluster (AN11582–AN7879).

A second approach is to explore known characteristics of the species in focus. In fungi, it is known that genes belonging to a single secondary metabolite pathway tend to cluster on the chromosome (Brakhage and Schroeckh, 2011). The *A. nidulans* information network includes the data of the chromosomal position of all genes. If two genes are neighbors, an edge is drawn between them. We applied Ondex' *genomic view* layout to immediately check the transcriptome data for the regulation of fungal gene clusters, because it lays out each chromosome separately and keeps the spatial information (see Figure 4). Differentially expressed gene clusters are recognized by the regions of the chromosome where several neighboring genes are depicted with larger glyphs (representing high fold-changes). This way, differentially expressed gene clusters could be identified in our analysis of the transcriptome data from Nahlik et al. (2010) (see Supplementary File S2).

**Figure 5:**
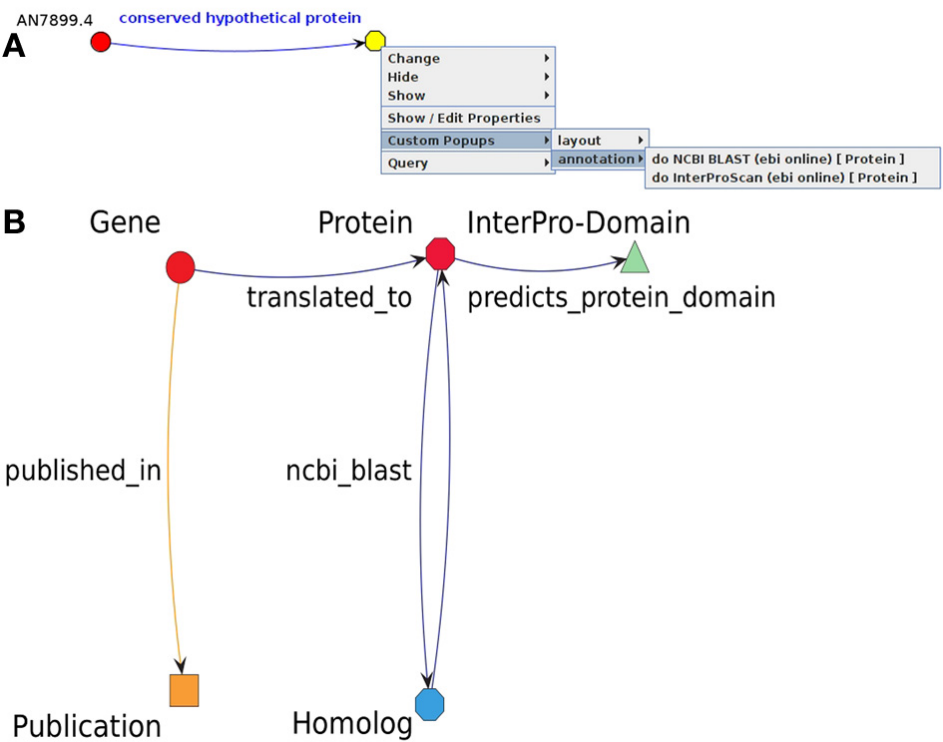
**Example application of the context-sensitive menu and meta-legend from the resulting network**. **(A)** Screenshot of context-sensitive menu available for the concept class *protein*. The protein concept of an associated gene may be either used to invoke an NCBI-BLAST or InterProScan protein domain prediction. **(B)** Meta-legend of resulting network which has been integrated using the customized context-sensitive menu. The gene names can be used to retrieve publications from PubMed Central or to download protein sequences from the BioMart web service. The protein sequence can be subsequently used to predict the protein domains with the help of InterProScan. Another possibility is to search for similar genes with the help of NCBI-BLAST. A backward BLAST is available in order to provide a bidirectional BLAST to make the prediction of homologs more reliable. All operations are only performed for selected nodes and use web services to integrate up-to-date data.

For *A. nidulans*, there were many genes with little or no information in the network. One way to enhance the completeness of the functional annotation is to consider gene annotations from orthologous genes for related species. Orthologous genes are known to likely have a similar function. The orthology information was integrated for the relatively well-characterized fungal species *A. fumigatus* and *S. cerevisiae*. Another approach is the retrieval of additional data from other databases or the prediction of gene functions by applying bioinformatics methods. Context-sensitive workflow items beneficial to the exploratory analysis of experimental data of the resulting network were developed (see Figure 5). The implemented methods use web services ensuring that the processed data is up to date while outsourcing computations to the service providers.

- **InterProScan**. The protein sequences of selected genes are retrieved from the BioMart web service (Kinsella et al., 2011). The sequence is used to predict protein domains by invoking the web service for InterProScan (Zdobnov and Apweiler, 2001). The retrieved protein domains and their corresponding information are added to the network.
- **Homolog search**. The protein sequences of selected genes are retrieved from BioMart web service (Kinsella et al., 2011). The web service for NCBI-BLAST (Altschul et al., 1990) is invoked with these sequences in order to search for similar sequences in UniProtKB (Magrane and UniProt Consortium, 2011). Significant results and their corresponding details are integrated into the network. Additionally, a bidirectional BLAST was implemented to check whether similar sequences were really homologous.
- **Full-text literature search**. Selected genes and their corresponding synonyms are used to search all available full-texts at PubMed Central. The metadata of publications is retrieved from the web service and subsequently integrated into the information network. It is used to download the full text, which itself is scanned for occurrences of any gene name and synonym which is present in the information network. The text bodies are pre-computed using suffix trees (Ukkonen, 1995), in order to allow high-speed text-search using many keywords in large texts. To connect the publication to the network, edges are drawn between the publication and any identified gene.

The application of these interactive menu items facilitated the on-the-fly retrieval of additional data as part of our analysis workflow. The resulting networks were laid out adequately with the *genomic view* layout, which is already part of the Ondex suite. All resulting networks have been manually checked if previously unobserved relations between the data concept lead to new hypotheses.

## 3. Results

**Figure 6:**
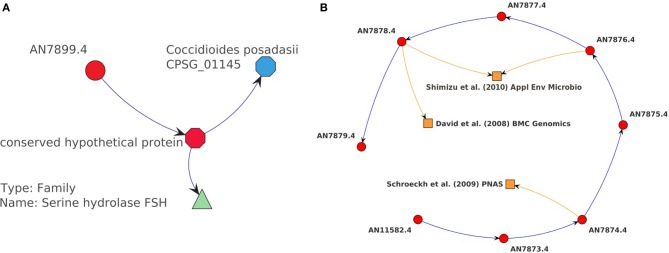
**Example annotation resulting from the usage of context-sensitive menus**. **(A)** A gene of interest (AN7899.4) is explored further by retrieving the protein sequence (CADANIAG00003918) from BioMart web service. This sequence is used to invoke the InterProScan which predicts a serine hydrolase domain. The protein sequence is also used to execute a bidirectional BLAST-search finding a potential homologous gene (CPSG_01145) which is involved in the secondary metabolism of *Coccidioides posadasii*. **(B)** A sequence of neighbored strongly differentially expressed genes (AN11582.4–AN7879.4) is selected to retrieve publications from the full-text search from PubMed Central. Three different publications are returned (orange squared glyphs).

In this study, we present new extensions of the Ondex system (Köhler et al., 2006) and demonstrate how they can be effectively applied to extract integrated information networks for new insights. We have enhanced the features within Ondex by implementing customizable context-sensitive menus which allow interactive integration of additional data while exploring the integrated information network in the Ondex front-end. The application of these context-sensitive menus enables interactive extensions of the network to be made by the user. This process is illustrated in Figures 3–6. The new functionality facilitates the gathering of additional information, which helps to retrieve existing annotations from web services and supports making hypotheses about possible gene functions. With the help of the interactive menus, we can overcome the strict separation between data integration and visualization. In this section, our approaches for the exploration of the specific data are presented. The precise workflow depends on intermediate results and the research focus of the experiment. To our knowledge, this is the first application of an integrative network analysis approach to *A. nidulans*.

### 3.1. Information network for *Aspergillus nidulans*

Data integration is especially important for less studied organisms, where often no reference genome data repositories such as Ensembl (Flicek et al., 2013) are available. Information networks function as the basis of the validation, prioritization, and selection of candidates from candidate lists resulting from modeling techniques. Usually, the gene annotation for less-studied organisms is highly fragmented and therefore it is necessary to call upon a large selection of less comprehensive resources in order to construct a representative annotation set. In order to expand the number of predicted functional annotations, it is common to integrate data from orthologous genes from closely-related species.

We applied Ondex to integrate publicly available data from *A. nidulans* (see Table 1). The resulting Ondex information network facilitates exploration of the existing data (see Figure 1). The network contains information for 10,527 genes, which are connected according to their chromosomal position. This allows for the detection and analysis of fungal chromosomal gene clusters. Furthermore, these genes were annotated with the three Gene Ontology (GO) domains: *biological process*, *cellular component*, and *molecular function* (Ashburner et al., 2000). The hierarchical structure of the Gene Ontology is preserved and 23,431, 3003, and 9397 different GO terms are integrated for each domain, respectively. Another set of functional annotations for *A. nidulans* is available from the Functional Catalog (FunCat) (Ruepp et al., 2004). This resource has more than 29,500 mappings between genes and 656 functional categories for *A. nidulans*. A large fraction of genes (2917) can also be mapped to pathways from the Kyoto Encyclopedia of Genes and Genomes (KEGG) (Kanehisa et al., 2012) and its corresponding hierarchical structure which contains 438 unique entities. Additionally, a genome-wide metabolic network model published by David et al. (2008) was integrated. This model incorporates 904 instances of enzymatic reactions being regulated by particular genes in *A. nidulans*. The whole metabolic network is comprised of 794 reactions and 738 metabolites. Pre-computed orthologous genes in *Aspergillus fumigatus* and *Saccharomyces cerevisiae* were also integrated from the Aspergillus Genome Database (AspGD) (Arnaud et al., 2010). The annotations of 2261 *S. cerevisiae* and 7054 *A. fumigatus* genes have contributed to filling in the gaps in the *A. nidulans* annotation in the instances where orthologous genes between these organisms and *A. nidulans* were identified. AspGD also provides manually curated occurrences of *A. nidulans* in 2123 publications, which were also imported and made available in the Ondex information network.

### 3.2. Integration of experimental data

Publicly available data for *A. nidulans* was integrated into an information network which was subsequently used to compare a wild-type and a Δ*csnE*-mutant at different developmental stages by re-analyzing published microarray data (Nahlik et al., 2010). After the integration of experimental data taken from Nahlik et al. (2010), it was possible to filter the network to display only nodes representing genes where the regulation was affected by the mutation. This processing facilitated the identification of general trends in the datasets.

A total number of 1252 genes were found to be differentially expressed due to the Δ*csnE* mutation, when only non-redundant gene identifiers were counted at all measurement points (see Table 2). The distribution of differentially expressed genes (DEGs) between different contrasts shows that, despite the fact that *csnE* is only expressed during the first vegetative growth phase, most changes in gene expression occur at later stages of sexual development after 48 h (see Table 2). (The term *contrast* refers to the comparison of transcript abundances between different conditions at certain time points, i.e., the Δ*csnE* mutant versus the wild-type at different developmental stages.) This implies that most changes caused by the mutant take place before cell differentiation. Specifically, 1161 genes (95.1% of all 1252 DEGs) have a |fold-change| ≥4 during sexual or asexual development in contrast to only 157 DEGs (12.5%) during vegetative growth. A similar proportion holds for higher |fold-changes| ≥8 (see Table 2). In fact, the effect of a gradually increased number of DEGs caused by the Δ*csnE*-mutation can already be observed by comparing the two different time points of the vegetative growth phase.

**Table 2:** **Number of differentially expressed genes for each growth stage**.

**Developmental stage**	**|**Fold-change**|** ≥ **4**	**|**Fold-change**|** ≥ **8**
V14	50	21
V20	134	45
V14 and V20	157	49
A48	980	438
S48	577	236
A48 and S48	1161	499
Total non-redundant DEGs	1252	530

In each analyzed growth stage (V14—vegetative growth after 14 h, V20—vegetative growth after 20 h, S48—sexual development after 48 h, A48—asexual development after 48 h), the wildtype is compared with a ΔcsnE mutant. The number of differentially expressed genes and strongly differentially expressed genes between both genotypes are shown.

### 3.3. Visual exploration of functional annotations

The network approach automatically considers that genes are mapped to different hierarchical levels of the functional annotation. As an example, in Figure 3B the gene AN3256.4 is associated with two different levels in the annotation hierarchy, i.e., the highest level *secondary metabolism* and a lower level *metabolism of phenylpropanoids*. In the pie chart visualization in Figure 3A, only the highest level is displayed and the gene is part of the section *secondary metabolism*. In order to show more detailed information about the lower hierarchies, new diagrams need to be drawn. The annotation-orientated network of each contrast forms a basis for further exploration, i.e., other functional annotation schemes such as GO, can be simultaneously shown and genes of interest can be displayed within the full context of all their annotated functional categories.

We performed a visual assessment of the functional annotation for all strongly differentially expressed transcripts at all four contrasts. Unlike Nahlik et al. (2010), we integrated publicly available functional annotations for *A. nidulans*, namely Functional Catalog and Gene Ontology. We tried to estimate whether our results (using automatically created data sources for the annotation) are comparable to the original publication (using manually assigned functional categories). The most prominent functional categories are *secondary metabolism*, *stress and defence related genes*, *cell wall* and genes associated with *transport* processes. In addition to the results published by Nahlik et al. (2010), a large proportion of differentially expressed genes is associated with *primary metabolism*. Due to the difference in the underlying functional annotation, the details of the results from the study of Nahlik et al. (2010) were not completely comparable. Nevertheless, our analysis could reproduce the main findings of the original publication, i.e., the set of mainly regulated functional categories and the observation that the largest transcriptomic changes occur after 48 h. This endorses the manual classification of the authors, as well as the one offered by publicly-available annotation resources based on ontologies.

An exploration of the distribution of functional annotations within the network provides a quick, intuitive overview of affected processes and forms the basis for further in-depth analyses of gene functions. It is an alternative to commonly used visualizations of functional annotations with the help of bar or pie charts (see Figure 3A) and provides a starting point for a more detailed data interpretation.

### 3.4. Exploration of fungal gene clusters

The genomic view provided by the integrated Ondex network allows gene clusters, which are *strongly* differentially expressed, to be easily identified (see Figure 4 and Supplementary File S2). Clusters of several strongly differentially expressed genes are possible indications for secondary metabolite gene clusters induced in the respective developmental stage.

Using the genomic view, the orsellinic acid synthesis cluster (AN7909–AN7914) is immediately identified as being strongly expressed in the vegetative growth phase after 20 h and in both developmental stages at 48 h (see Figure 4). These genes have been subject to the newly added interactive function—carrying out a full-text literature at PubMed Central. The genes that are part of the orsellinic acid cluster are linked to publications that have investigated their gene function. Although the precise function of products of this gene cluster is unknown, it has been shown that it is expressed if *A. nidulans* is co-cultured with the actinobacteria *Streptomyces rapamycinicus*, which is found in the same biological habitat (Schroeckh et al., 2009). The bacteria induces the expression of the orsellinic acid by histone modifications; in particular through the main histone acetyltransferase complex Saga/Ada (Nützmann et al., 2011). This gene cluster is therefore proposed to be part of a signalling pathway, which is involved in the communication between microbes of different species. The published data suggests that the interplay between fungi and microbes might be connected to the fungal development via the signalosome complex of *A. nidulans*. The exploration of the information network with Ondex linked this experiment, investigating the fungal signalosome, to publications focusing on fungal-bacterial interaction.

A second differentially expressed gene cluster of high interest was the characterized sterigmatocystin biosynthesis pathway which is composed of 25 genes (AN7805–AN7825) and located on chromosome IV (Brown et al., 1996) (see Supplementary File S2). This gene cluster is only expressed during the asexual growth stage, where large amounts of intermediate metabolites of this chemical structure have been verified by Nahlik et al. (2010). They possibly result from an inhibited secretion of the metabolite into the medium. The regulation of this gene cluster is very important since sterigmatocystin contributes to the defence of the cell against other microorganisms in the same habitat during this developmental phase. A more detailed functional annotation of this gene cluster was undertaken using our newly developed context-sensitive workflows for the Ondex system.

### 3.5. Gene annotation using the context-sensitive menus

Gene clusters found in the previous step were explored in greater detail in order to confirm their relevance. At this stage, several context-sensitive menus were used to obtain additional annotations (see Figure 5). As gene clusters encode for a single functional unit, genes encoding for particular enzymes, pathway regulators, and related transporters should be near each other on the chromosome in fungi. A hypothesis about the function of genes surrounding the sterigmatocystin gene cluster (AN7805–AN7825) was formed using Ondex. The InterProScan web service was invoked for neighboring genes surrounding the cluster. The observation that the neighboring gene AN7797.4 is highly down-regulated (fold-change = −3.25 and adjusted *P*-value < 10^−7^) and the prediction of a transmembrane protein domain by InterProScan led us to conjecture that this is a potential sterigmatocystin transporter, which needs to be validated experimentally (data not shown).

Another example of our exploratory data analysis was the application of the InterProScan for gene AN7899.4, in the region of the predicted NRPS AN7884.4 and the already described NRPS-PKS AN7909.4 (see Figure 6A). The gene is strongly differentially expressed in the mutant during vegetative growth after 20 h (fold-change = 20.25 and adjusted *P*-value < 10^−5^). The InterProScan predicts that this gene contains a serine hydrolase domain and is therefore catalytically active. Additionally, the protein sequence was used to invoke a BLAST web service in order to search for potential similar genes. We found that only the CPSG_01145 gene from the pathogenic fungus *Coccidioides posadasii* had a high sequence similarity of more than 60%. It is annotated as citrinin biosynthesis oxidoreductase CtnB and therefore is likely to be involved in the secondary metabolism of this fungus. These findings and the chromosomal location of AN7899.4 in the proximity of two secondary metabolite gene clusters has not been reported before and make this gene interesting for further experimental research. The example shows that the added Ondex functionality helps to make new hypotheses which otherwise would not have been recognized.

Literature data is the most reliable and abundant source of information, which is regularly updated. The full text of a large fraction of publications can be mined with the help of the PubMed Central database web service. We were able to take advantage of this functionality using the context-sensitive menu system developed to support this application case. The full-text search was executed for all papers relating to a gene cluster (AN11582–AN7879) which was strongly differentially expressed during the asexual growth stage (see Figure 6B). This cluster was of great interest due to its close location to the orsellinic acid gene cluster. For three genes, an associated publication was found. A more detailed inspection revealed that in the paper by Shimizu et al. (2010) AN7876.4 and AN7878.4 were predicted to encode the transaminase B genes, whereas in the paper by Schroeckh et al. (2009) it was found that this gene cluster is co-expressed with the orsellinic acid gene cluster during co-cultivation with *S. rapamycinicus*. This approach shows that our extension easily reveals and visualizes connections between different studies which supports data interpretation. In contrast to a sole analysis in a web browser, Ondex instantly integrates newly found citations within the information network. The new concepts can form the basis for further additional data integration. That way, it is much easier to reproduce the same chain of reasoning. Additionally, the new network can benefit from other basic functionalities of Ondex, i.e., interactive or automatic visualization of the information network, the creation of additional labels, and the usage of filters. Overall, the integration of new knowledge into the networks ensures that it is possible to keep track of different data sources and the connection between them.

In summary, the custom workflows for *A. nidulans* are a proof-of-concept for our extensions to the Ondex framework. The user-defined context-sensitive menus provide new functionality that makes better use of existing features of Ondex. As the import of additional data is controlled by the user and can be limited to particular sub-networks, this approach helps to address the scalability problem when working with large datasets. Eventually, this procedure leads to a lower overall memory usage of Ondex. The implementation of the new functionality within Ondex emphasizes high cohesion, low coupling, and encapsulation, thus ensuring the re-usability of the code. Individual menu items can be seen as add-on elements, easily allowing the Ondex functionality to be extended in a modular way. An integrated editor allows easy implementation of new menus, which can be adapted for the specific data and analysis requirements.

Our extensions to the Ondex data integration and visualization framework improves its applicability for exploratory data analysis. The presented context-sensitive workflows extend the functionality of Ondex and helped to propose new interpretations of experimental data. Although the presented data integration scheme is tailored for the interpretation of the gene expression data in the context of secondary metabolite analysis, this framework and the presented workflows will benefit the analysis of other datasets. Data can be interactively visualized and additional data can be integrated on demand. Thus, the user is not limited to a pre-defined analysis workflow and to previously integrated data. At the same time, the advantages of computer-assisted data integration and visualizations are retained.

## 4. Discussion

The quality of network inference models does not only need to be assessed with the help of quantitative models but the resulting network topology also needs to be evaluated qualitatively. Currently, the qualitative assessment requires in-depth expert knowledge about the components in the network model and about its dependence upon the experimental setting. Online resources providing static, pre-integrated knowledge, such as BiologicalNetworks (Kozhenkov et al., 2011) and GeneMania (Mostafavi et al., 2008), focus on widely-studied model organisms or require the upload of experimental in-house data. With the increasing number of sequenced organisms, we predict a further diversification of studied organisms and an increased need to create custom integration networks. Thus, the application and improvement of data integration and visualization software providing the possibility to compose integrated datasets using custom workflows according to user specifications is essential. Currently, the utilization of such tools is hampered by the challenge of proper data integration and visualization for large datasets. Here, we describe the extension of the data integration and visualization framework Ondex allowing the user to build context-sensitive workflows. The workflows described here are examples of an exploration of experimental results followed by a more detailed analysis, which have led to new hypotheses about the functions of currently unannotated genes. The strength of our approach is that it captures the essential information from a complex network of integrated publicly available data while the analysis can be individually tailored for each network region of interest in order to reduce the overall computational effort. In the manner of an exploratory data analysis, the workflow can be easily adjusted by the scientist to develop innovative research questions and identify patterns within the data that emerge from a combination of analysis and expert judgement. The provision of data integration functionalities with the help of pop-up menus is convenient and intuitive. This way, the graphical interface does not need additional separate windows and the researcher does not have to become acquainted with a specialized user interface, e.g., via a scripting language.

We used Ondex to integrate publicly-available key datasets for *A. nidulans*, which would otherwise be spread over different resources. The mapping of the information allows the data to be easily explored and visualized in an intuitive manner. This network can then serve as a scaffold for further integration of additional experimental data. The information connected to a gene locus helps the user to confirm predictions and generate hypotheses. In the case where comprehensive data about a gene set of interest is missing, context-sensitive menus can be applied for the prediction of gene functions. Therefore, Ondex can help to steer the selection of new experiments and define new directions for further investigation. This is the first integrative approach based on networks applied to *A. nidulans*. This study demonstrates the exploration of co-expressed gene clusters for secondary metabolite biosynthesis pathways. The exploratory analysis helped to link the data to other publications covering a fungal-bacteria interaction. It also enabled the identification and annotation of differentially expressed genes in the proximity of gene clusters. The uncharacterized gene AN7797.4 may be a transporter involved in the sterigmatocystin pathway, whereas the uncharacterized gene AN7899.4 may be part of the metabolic pathway of the orsellinic acid. It needs to be explored in further experiments whether and how these genes are directly involved in the regulation of these clusters.

Our proposed procedure to analyze gene expression data with the focus on fungal secondary metabolite gene clusters could have been performed without the assistance of the Ondex framework. In a traditional approach, we would have filtered the interesting differentially expressed genes in spreadsheets resulting from statistical analysis. Afterwards, additional information would be gathered using a web browser. Different resources such as genome browsers, the online InterProScan tool, the online BLAST tool, and the PubMed Central search interface need to be consulted. These research steps need to be performed in succession for each gene of interest separately while keeping in mind that each gene may have different gene identifiers (which is especially important for performing a full-text literature search). By providing these data integration functionalities through context-sensitive menus in the Ondex framework, the data interpretation procedure is sped up, it is more reproducible, and it helps to direct the researcher's focus on the data interpretation rather than the methodology of retrieving it. Another advantage of the approach to data integration supported by the Ondex framework is that it facilitates tracking of different sources of data and the path of reasoning and exploration. This would not be possible using web resources and their interfaces alone, which work mostly in a sequential, linear manner. All publicly available information about a gene can be consolidated within one graph, making the navigation easier and ensuring the best possible quality of data, as all relevant data can be efficiently collected. If the information about a gene locus is missing, the utility of Ondex to draw conclusions is limited and additional experimental data or bioinformatic methods are necessary to fill the gap. Thus, the completeness of the underlying functional annotation is of particular importance as it has a major impact on the subsequent interpretation of the dataset. In our example, it became apparent that most conclusions about the functional categorization of *A. nidulans* genes can be drawn from the Functional Catalog, which has already been successfully applied to fungal genes and proteins in other studies (Priebe et al., 2011). Ondex visualization ensured that additional information provided from GO was not disregarded at any point during analyses.

The standard procedure in Ondex is to integrate available data from different data sources prior to the visualization and data analysis. If a large amount of data is integrated, it results in large datasets which need to be handled and visualized by the Ondex software framework. Currently, the complexity of layout algorithms and the computational limitations, i.e., memory or CPU, make it challenging to manage the vast amount of data in a user-friendly and responsive manner. Additionally, if access to the most recent information is very important and the underlying data changes frequently, the time-consuming step of data integration has to be repeated regularly. If the data originates from user-made, computationally demanding calculations, a frequent data integration becomes computationally infeasible. Our extension of the Ondex framework overcomes these limitations by offering the option to apply these steps to a selected part of the network via the context-sensitive menus. Thus, the required amount of data is reduced, current data can be instantly downloaded from web resources, and intensive calculations need only be performed for subsets of the available data relevant to the current focus of investigation. Hence, the memory and computational load is reduced and access to the most recent data is guaranteed.

In this way, the context-sensitive menus make the interactive data analysis more efficient and user-friendly by providing data integration and filtering on-the-fly. The precise workflow of data analysis does not have to be established for the whole data integration process beforehand and the integration can be repeatedly applied and adjusted during the interactive analysis. This extension to the Ondex framework now combines the advantages of two data integration paradigms, i.e., of data warehousing and federated data integration, into one easy-to-use single system. The extensions to Ondex reported here have significantly improved its suitability for its usage for the qualitative assessment of inferred network models.

## Author contributions

Fabian Horn initiated and led the work presented here, contributed as the main author to this manuscript and carried out the analysis. Martin Rittweger implemented the context-sensitive menus in Ondex and carried out the analysis. Jan Taubert supported the general implementation in Ondex and contributed to conceptualization and writing of the manuscript. Artem Lysenko supported the implementation with respect to Ondex scripting capabilities and provided feedback on the manuscript. Christopher Rawlings and Reinhard Guthke supervised the work at Rothamsted and the HKI, respectively, and provided feedback on the manuscript. All authors have read and acknowledged the manuscript.

### Funding

This study was supported by the International Leibniz Research School for Microbial and Molecular Interactions (ILRS), as part of the excellence graduate school Jena School for Microbial Communication (JSMC), supported by the Deutsche Forschungsgemeinschaft. The work on Ondex has been funded by BBSRC Grants BBS/B/13640 and BB/F006039/1.

### Conflict of interest statement

The authors declare that the research was conducted in the absence of any commercial or financial relationships that could be construed as a potential conflict of interest.

## Supplementary material

Supplementary File S1 **Functional Annotation of Differentially Expressed Genes.** A spreadsheet which contains the number of differentially expressed genes for each Functional Catalog (FunCat) category. In order to make the results more comparable to the original publication by Nahlik et al. (2010), the categories are merged. The merging rules and the resulting numbers are given.

Supplementary File S2 **List of Strongly Differentially Expressed Gene Clusters.** For each growth stage, the clusters and their associated gene identifiers are listed (|fold-change| ≥8 and adjusted *P*-value ≤0.05). An annotation is given if the cluster is known or predicted to be part of fungal secondary metabolism.
